# Long-term Changes in the Premature Death Rate in Lung Cancer in a Developed Region of China: Population-based Study

**DOI:** 10.2196/33633

**Published:** 2022-04-20

**Authors:** Wenjing Ye, Weiwei Lu, Xiaopan Li, Yichen Chen, Lin Wang, Guangwang Zeng, Cheng Xu, Chen Ji, Yuyang Cai, Ling Yang, Zheng Luo

**Affiliations:** 1 Department of Respiratory Medicine Xinhua Hospital Shanghai Jiao Tong University School of Medicine Shanghai China; 2 Department of General Practice Xinhua Hospital Shanghai Jiao Tong University School of Medicine Shanghai China; 3 Department of Health Management Center Zhongshan Hospital Shanghai Medical College of Fudan University Shanghai China; 4 Office of Scientific Research and Information Management Pudong Institute of Preventive Medicine Shanghai China; 5 Center for Disease Control and Prevention Pudong New Area Shanghai China; 6 Department of Geriatrics Xinhua Hospital Shanghai Jiao Tong University School of Medicine Shanghai China; 7 Institute of Hospital Development Strategy China Hospital Development Institute Shanghai Jiao Tong University Shanghai China; 8 Warwick Clinical Trials Unit Warwick Medical School Coventry United Kingdom; 9 School of Public Health Shanghai Jiao Tong University School of Medicine Shanghai China; 10 Shanghai University of Medicine & Health Sciences Affiliated Zhoupu Hospital Pudong New Area Shanghai China

**Keywords:** lung cancer, mortality, years of life lost, trend analysis, decomposition method

## Abstract

**Background:**

Lung cancer is a leading cause of death worldwide, and its incidence shows an upward trend. A study of the long-term changes in the premature death rate in lung cancer in a developed region of China has great exploratory significance to further clarify the effectiveness of intervention measures.

**Objective:**

This study examined long-term changes in premature lung cancer death rates in order to understand the changes in mortality and to design future prevention plans in Pudong New Area (PNA), Shanghai, China.

**Methods:**

Cancer death data were collected from the Mortality Registration System of PNA. We analyzed the crude mortality rate (CMR), age-standardized mortality rate by Segi’s world standard population (ASMRW), and years of life lost (YLL) of patients with lung cancer from 1973 to 2019. Temporal trends in the CMR, ASMRW, and YLL rate were calculated by joinpoint regression expressed as an average annual percentage change (AAPC) with the corresponding 95% CI.

**Results:**

All registered permanent residents in PNA (80,543,137 person-years) from 1973 to 2019 were enrolled in this study. There were 42,229 deaths from lung cancer. The CMR and ASMRW were 52.43/10^5^ and 27.79/10^5^ person-years, respectively. The YLL due to premature death from lung cancer was 481779.14 years, and the YLL rate was 598.16/10^5^ person-years. The CMR and YLL rate showed significantly increasing trends in men, women, and the total population (*P*<.001). The CMR of the total population increased by 2.86% (95% CI 2.66-3.07, *P*<.001) per year during the study period. The YLL rate increased with an AAPC of 2.21% (95% CI 1.92-2.51, *P*<.001) per year. The contribution rates of increased CMR values caused by demographic factors were more evident than those caused by nondemographic factors.

**Conclusions:**

Lung cancer deaths showed an increasing trend in PNA from 1973 to 2019. Demographic factors, such as the aging population, contributed more to an increased CMR. Our research can help us understand the changes in lung cancer mortality and can be used for similar cities in designing future prevention plans.

## Introduction

Lung cancer is the leading cause of cancer-related deaths worldwide, with 2,206,771 new lung cancer cases and 1,796,144 deaths in 2020 [1]. Many studies have made tremendous efforts in the discovery of potential biomarkers for the detection, classification, and progression monitoring of lung cancer. Lung cancer treatment has made great progress over the past decade. Study of the long-term changes in the disease burden of lung cancer has great exploratory significance to further clarify the epidemiological characteristics of lung cancer and improve patient survival times [2].

In recent decades, China has witnessed rapid economic development, and tremendous changes have occurred in the population and epidemics [3]. Shanghai is an economic, science and technology, industrial, financial, and exhibition center and is 1 of the earliest cities in China to enter an aging society. Shanghai is a representative city for modernization development in China. In the next 20 years, other cities in China and other low- and middle-income countries (LMICs) are likely to follow the development characteristics of Shanghai [4]. The Shanghai Pudong New Area (PNA) officially began to develop on April 18, 1990. Since then, Pudong has become 1 of the areas with the fastest urbanization and economic growth in China. Economic growth and total scale have highlighted the speed and height of Pudong’s development and opening up. Over the past 30 years, PNA has adhered to reform, expansion, and strengthened innovation. It has developed from a field into a modern urban area with concentrated elements and advanced facilities. It has become the epitome of Shanghai's modernization and the symbol of China's reform and expansion [5,6].

Cancer is a disease that seriously endangers human health. In the past decade, cancer has been the main cause of death in China [7]. The treatment of cancer has become the most important research direction in the world to improve life expectancy [1]. Years of life lost (YLL) refers to the loss of life caused by early death. It can more accurately reflect the burden on society [7]. The aging population, progress of treatment, smoking, and environmental pollution may be factors that affect long-term changes in the premature death rate in lung cancer. An epidemiological study of lung cancer mortality trends over time may help quantify its impacts on public health and society, promote the assessment of current protocols for lung cancer, and define high-risk populations that will benefit from early detection programs for lung cancer [8]. Our study examined long-term changes in the premature death rate in lung cancer from 1973 to 2019 in PNA, Shanghai, China, to improve public health.

## Methods

### Data Source

Cancer death data were collected from the Mortality Registration System of PNA, including age, gender, date, and cause of death. The complete population data were provided by the Statistics Bureau and the Public Security Bureau of PNA [9]. Periodic evaluations, data cleaning, and compilation were performed to ensure completeness of the registration system. The per capita gross domestic product (GDP) of Shanghai and PNA were collected from the Shanghai Municipal Bureau of Statistics [10] and the Shanghai PNA Bureau of Statistics [11].

Deaths from malignant neoplasm of the trachea (C33) and bronchus and lung (C34) were classified by the underlying cause of lung cancer deaths according to the *International Classification of Diseases 10th Revision* (ICD-10) [12]. Since the data covered a long time span of 47 years, data before 1975, coded based on ICD-8, and data for 1975-2001, coded based on ICD-9, were converted to ICD-10 codes.

Causes of death were coded by rigorously trained clinicians, and each record was further verified by the Center for Disease Control and Prevention (CDC).

### Ethics Approval and Consent to Participate

Our study did not involve any intervention in human participants. The surveillance protocol was approved by the ethical committee of the Shanghai PNA Center CDC. Strict confidentiality of individual data was practiced throughout the study.

### Statistical Analyses

The crude mortality rate (CMR) and the age-standardized mortality rate by Segi’s world standard population (ASMRW) of neurological disorders were calculated per 100,000 (/10^5^). The CMR and ASMRW between genders were compared using the Poisson approximation method and the Mantel-Haenszel test, respectively.

The YLL was calculated according to the original method described by Murray and Lopez [13]. The equation used to calculate the YLL is as follows [14]:

YLL = KCe^ra^/(r + β)^2^｛e^–( r + β)(L + a)^ [–( r + β)(L + a) – 1] – e^–(r + β)a^[–(r + β)a – 1]} + [(1 – k)/r] × (1 – e^–rL^),

where a is the age at death, *β* is the age weighting parameter (*β*=.04), C is the age weighting fit with constant (C=0.1658), r is the discount rate (r=3%), L is the standard life expectancy at the age of death according to the standard reference life table for the Global Burden of Disease (GBD) study [15], and e is the Napier constant.

The calculation of the YLL was performed using the World Health Organization (WHO) template [14].

Temporal trends in the CMR, ASMRW, and YLL rate were calculated using joinpoint Regression 4.3.1.0 (National Cancer Institute, Bethesda, MD, USA) and expressed as an average annual percentage change (AAPC) with a corresponding 95% CI. The Z test was performed to assess whether the AAPC was statistically different from 0. The terms “increase” and “decrease” were used to describe a statistically significant (*P*<.05) AAPC, while “stable” was used for not statistically significant trends.

Age was classified into 8 groups: 0-4, 5-14, 15-29, 30-44, 45-59, 60-69, 70-79, and 80+ years. Age-specific CMRs were calculated for each age group. Changes in the mortality rates of each period in 5 years from 1973 to 2019 were compared with the period before it or the data from 1973 to 1979, and causes from demographic and nondemographic factors were estimated by the decomposition method, in which mortality rates were calculated and compared for each 5-year age group, from 0-4 to 85+ years [16]. All statistical analyses were conducted using SPSS Statistics version 21.0 (SPSS, Inc, Chicago, IL, USA) and R version 3.4.3 (R Core Team). Statistical significance was set at *P*<.05.

## Results

### Baseline Characteristics of Underlying Death from Lung Cancer

From 1973 to 2019, all registered permanent residents in PNA, with a total of 80,543,137 person-years, were enrolled in this study. There were 42,229 deaths from lung cancer. Of these, 30,638 (72.55%) patients were men. The median age at death from lung cancer was 72.10 years, and the average age at death was 70.96±11.21 years. The CMR and ASMRW of lung cancer were 52.43/10^5^ and 27.79/10^5^ person-years, respectively. In addition, the CMR and ASMRW were 77.04/10^5^ and 44.27/10^5^ person-years, respectively, in men, while the corresponding rates were 28.43/10^5^ and 13.77/10^5^ person-years, respectively, in women (Table 1).

**Table 1:** Baseline characteristics of deaths (1973-2019).

Characteristic			Deaths, n (%)		Age (years), mean (SD)		Age (years), median		CMR^a^ (/10^5^ person-years)		ASMRW^b^ (/10^5^ person-years)		YLL^c^ (years)		YLL rate (/10^5^ person-years)
**Gender**															
	Male	30,638 (72.55)		70.54 (10.74)		71.56		77.04		44.27		343,728.73		864.30	
	Female	11,591 (27.45)		72.08 (12.30)		73.73		28.43		13.77		138,050.40		338.58	
**Period**															
	1973-1979	929 (2.20)		64.88 (10.31)		66.38		21.57		23.40		12,915.91		299.88	
	1980-1984	969 (2.29)		65.75 (11.14)		67.08		29.29		28.79		13,006.25		393.17	
	1985-1989	969 (2.29)		66.47 (10.98)		67.48		31.51		28.45		12,711.93		413.33	
	1990-1994	1890 (4.48)		67.24 (10.42)		68.18		40.02		31.10		24,095.98		510.23	
	1995-1999	4797 (11.36)		68.32 (10.70)		69.77		43.16		30.70		59,014.12		530.92	
	2000-2004	6363 (15.07)		69.73 (11.10)		71.43		52.98		31.23		74,513.88		620.42	
	2005-2009	7595 (17.99)		71.22 (11.33)		73.46		57.88		27.68		84,179.23		641.55	
	2010-2014	8832 (20.91)		71.66 (11.47)		73.34		63.10		26.28		96,872.05		692.14	
	2015-2019	9885 (23.41)		72.65 (10.85)		72.62		66.40		24.02		104,535.93		702.19	
Total			42,229 (100.00)		70.96 (11.21)		72.10		52.43		27.79		481,779.14		598.16

^a^CMR: crude mortality rate.

^b^ASMRW: age-standardized mortality rate by Segi’s world standard.

^c^YLL: years of life lost.

### Age-specific Mortality in Lung Cancer

The CMRs in the age groups of 0-4, 5-14, 15-29, 30-44, 45-59, 60-69, 70-79, and ≥80 years were 0.09/10^5^, 0.02/10^5^, 0.36/10^5^, 4.26/10^5^, 35.97/10^5^, 132.70/10^5^, 302.76/10^5^, and 372.98/10^5^ person-years, respectively (Table 2).

**Table 2:** Number of deaths, CMR^a^, YLL^b^, and YLL rates (1973-2019).

Group by age (years)		Deaths, n (%)	CMR (/10^5^ person-years)	YLL (years)	YLL rate (/10^5^ person-years)	
**Age (years)**						
	0-4	3 (0.01)	0.09	90.93	2.61	
	5-14	1 (0.002)	0.02	58.54	0.73	
	15-29	57 (0.13)	0.36	1543.65	9.62	
	30-44	826 (1.96)	4.26	19,559.72	100.95	
	45-59	6385 (15.12)	35.97	119,690.09	674.25	
	60-69	11,521 (27.28)	132.70	159,523.69	1837.34	
	70-79	14,642 (34.67)	302.76	134,731.32	2785.91	
	≥80	8793 (20.82)	372.98	46,581.20	1975.87	
Total		42,229 (100.00)	52.43	481,779.14	598.16	

^a^CMR: crude mortality rate.

^b^YLL: years of life lost.

### Burden of Premature Death from Lung Cancer

From 1973 to 2019, the YLL due to premature death from lung cancer was 481779.14 years and the YLL rate was 598.16/10^5^ person-years. The YLL and rate of YLL in men (343,728.73 years and 864.30/10^5^ person-years, respectively) were higher than those in women (138,050.40 years and 338.58/10^5^ person-years, respectively); see Table 1. In terms of age, the top 3 YLL were in the age groups of 60-69, 70-79, and 45-59 years, which were 159,523.69, 134,731.32, and 119,690.09 years, respectively. The top 3 YLL rates were in the age groups of 70-79, 80+, and 60-69 years, which were 2785.91/10^5^, 1975.87/10^5^, and 1837.34/10^5^, respectively (Table 2).

### Trends in Mortality and YLL in Lung Cancer

**Figure 1:**
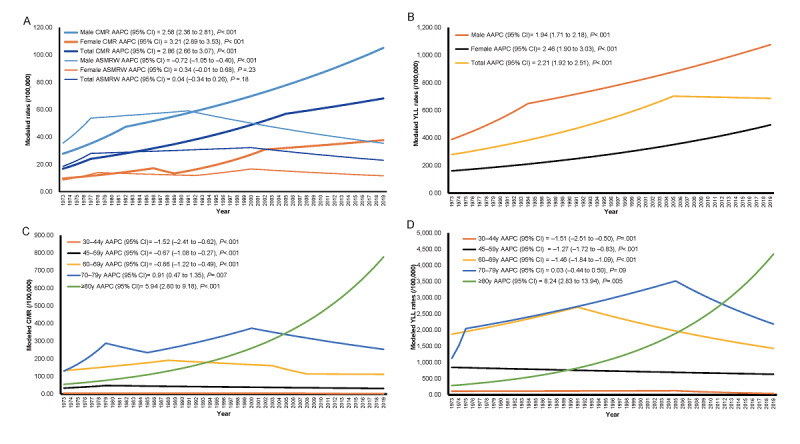
Trends in the CMR (/10^5^ person-years), ASMRW (/10^5^ person-years), and YLL (/10^5^ person-years) rate of persons dying from lung cancer by pathology type and age group in Shanghai PNA from 1973 to 2019. (A) CMR and ASMRW in lung cancer, (B) YLL rate in lung cancer, (C) CMR of age groups, and (D) YLL rate of age groups. AAPC: average annual percentage change; ASMRW: age-standardized mortality rate by Segi’s world standard population; CMR: crude mortality rate; PNA: Pudong New Area; YLL: years of life lost.

The temporal trends in the CMR, ASMRW, and YLL rate were expressed based on the modeled CMR, ASMRW, and YLL rate, as shown in Figure 1. The CMR and YLL rate for deaths from lung cancer showed significantly increasing trends in men and women, and the total population during 1973-2019 (all *P*<.001). The ASMRW decreased in men by 0.72% (95% CI –1.05 to –0.40, *P*<.001) per year, while the ASMRW in women and the total population during 1973-2019 was not statistically significant (*P*=.23 and .18, respectively). The CMR in lung cancer in the total population increased by 2.86% (95% CI 2.66-3.07, *P*<.001) per year during the study period. The YLL rate increased with an AAPC of 2.21% (95% CI 1.92-2.51, *P*<.001) per year from 1973 to 2019 (Figures 1A and 1B).

Regarding age-specific mortality, the YLL, CMR, and ASMRW of the total population were observed from 1973 to 2019 (Figures 1C and 1D). The increasing trends in the CMR were also observed in the age groups of 70-79 years (*P*=.01) and 80+ years (*P*<.001). The 30-44-, 45-59-, and 60-69-year age groups had statistically decreasing trends in the CMR (*P*<.001). The YLL rate increased by 8.24% (95% CI 2.83-13.94, *P*=.01) per year in the age group of 80+ years and 0.03% (95% CI –0.44 to 0.50, *P*=.09) per year in the age group of 70-79 years. However, the YLL rate decreased by 1.51% (95% CI –2.51 to 0.05, *P*=.001) per year in the age group of 30-44 years, 1.27% (95% CI –1.72 to 0.83, *P*<.001) per year in the age group of 45-59 years, and 1.46% (95% CI –1.84 to 1.09, *P*<.001) per year in the age group of 60-69 years (Figures 1C and 1D).

### Quantitatively Impacts of Demographic and Nondemographic Factors on Increased CMRs

**Figure 2:**
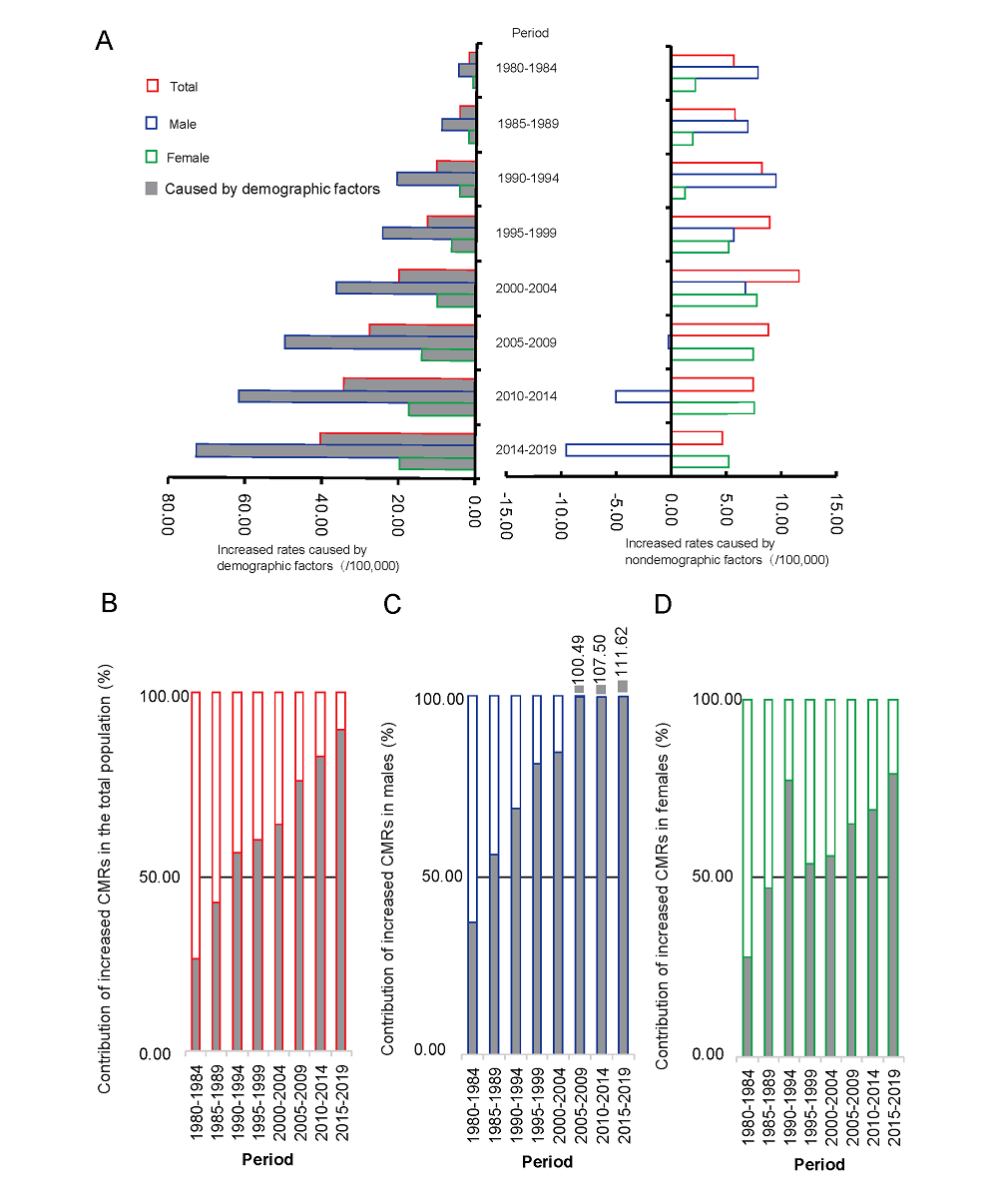
Increased CMRs caused by demographic and nondemographic factors and their proportion during the period from 1973 to 2019 compared with the CMR in lung cancer during 1973-1979 in Shanghai PNA. (A) Increased CMRs, (B) contribution of increased CMRs in the total population, (C) contribution of increased CMRs in men, and (D) contribution of increased CMRs in women. CMR: crude mortality rate; PNA: Pudong New Area.

The increasing CMRs caused by nondemographic and demographic factors are shown in Figure 2. Based on the CMR in lung cancer in 1973-1979, no statistically significant trend was found caused by nondemographic factors in the total population, with an AAPC of 0.17% (95% CI –11.34 to 13.16, *P*=.97) from 1980 to 2019, but a significant upward trend was observed in the increased CMR caused by demographic factors (AAPC [95% CI]=51.70% [35.48-69.88], *P*<.001). In men, the increased CMR caused by nondemographic factors decreased by 32.96% (95% CI –51.68 to –6.99, *P*=.02) during 1980-2019, and the CMR caused by demographic factors increased by 46.42% (95% CI 32.23-62.03, *P*<.001). In women, the increased CMR caused by nondemographic factors showed an upward trend with an AAPC of 24.24% (95% CI 2.60-50.44, *P*=.03), and the CMR caused by demographic factors also increased (AAPC [95% CI]=55.63% [38.54-74.83], *P*<.001); see Table 3. Figure 2B-D shows the proportion of increased CMR values caused by nondemographic and demographic factors. From 1985 to 2019, demographic factors played a decisive role in the contribution of the CMR compared to 1973-1979.

**Table 3:** Increased CMRs^a^ caused by demographic and nondemographic factors and their contribution during the period from 1973 to 2019 compared with the CMR in lung cancer during 1973-1979 or the period before it in Shanghai PNA^b^.

Comparison periods		CMR of the based period (/10^5^ person-years)	CMR of the other period (/10^5^ person-years)	*D* value of mortality (/10^5^)	Impact of demographic factors			Impact of nondemographic factors	
					Added value (/10^5^ )	Contribution rate (%)	Added value (/10^5^)		Contribution rate (%)
**Panel A** (based on the first period)									
	1980-1984 vs 1973-1979	21.57	29.29	7.72	2.00	25.68	5.73		74.14
	1985-1989 vs 1973-1979	21.57	31.51	9.94	4.11	41.38	5.83		58.62
	1990-1994 vs 1973-1979	21.57	40.02	18.45	10.20	55.28	8.25		44.72
	1995-1999 vs 1973-1979	21.57	43.16	21.59	12.70	58.83	8.89		41.17
	2000-2004 vs 1973-1979	21.57	52.98	31.41	19.86	63.21	11.55		36.79
	2005-2009 vs 1973-1979	21.57	57.88	36.31	27.45	75.58	8.87		24.42
	2010-2014 vs 1973-1979	21.57	63.10	41.53	34.10	82.11	7.43		17.89
	2015-2019 vs 1973-1979	21.57	66.40	44.83	40.24	89.76	4.59		10.24
**Panel B** (based on the last period)									
	1980-1984 vs 1973-1979	21.57	29.29	7.72	2.00	25.68	5.73		74.14
	1985-1989 vs 1980-1984	29.29	31.51	2.22	2.66	114.33	–0.44		-14.33
	1990-1994 vs 1985-1989	31.51	40.02	8.51	7.61	89.34	0.91		10.66
	1995-1999 vs 1990-1994	40.02	43.16	3.14	2.72	86.91	0.41		13.09
	2000-2004 vs 1995-1999	43.16	52.98	9.82	8.13	82.79	1.69		17.21
	2005-2009 vs 2000-2004	52.98	57.88	4.90	10.12	134.01	–5.21		34.01
	2010-2014 vs 2005-2009	57.88	63.10	5.22	7.86	125.16	–2.64		25.16
	2015-2019 vs 2010-2014	63.10	66.40	3.30	7.80	136.61	–4.50		36.61

^a^CMR: crude mortality rate.

^b^PNA: Pudong New Area.

## Discussion

### Principal Findings

It is crucial to understand the long-term changes in the rate of premature death from lung cancer for medical treatment research to formulate future preventive measures. The goal of “Healthy China 2030” has been to reduce the premature death rate of noncommunicable diseases by 30% [17]. Since 2000, many cities in China have gradually entered an aging society, and Shanghai is the first to do so. In recent years, the aging in Shanghai has not been alleviated but has gradually increased. Since 2018, PNA as a miniature Shanghai has already entered a superaging society, with a proportion of over 20% (Multimedia Appendix 1). We concluded that the increasing trends in the CMR were seen in the age groups of 70-79 years (*P*=.01) and 80+ years (*P*<.001) in terms of age-specific mortality and burden. The YLL rate increased by 8.24% (95% CI 2.83-13.94, *P*=.01) per year in the age group of 80+ years and 0.03% (95% CI –0.44 to 0.50, *P*=.09) per year in the age group of 70-79 years. The proportion of individuals aged 70-79 years (Multimedia Appendix 2) was almost the largest since 1995. The age groups under 70 years had statistically decreasing trends in lung cancer CMR (*P*<.001). It is evident that aging has contributed to an increase in lung cancer mortality.

Economic development, such as an increased GDP, improves public health. However, some factors related to the developed economy, such as lifestyle factors and environmental and medical levels, may also influence the mortality rate. Globally, smoking-attributable deaths have increased by 20.1% (15.3-25.2) since 1990, with most deaths occurring in China [18]. In China, smoking has either peaked or continued to increase [19]. According to our statistics over the past 50 years, the YLL rate increased with an AAPC of 2.21% per year from 1973 to 2019, and this may be associated with the increased smoking rate. The YLL rate of men is higher than that of women (Figure 1), which is consistent with the difference in the smoking proportion between men and women.

Outdoor air pollution exposure is a clear carcinogen to humans [20]. For decades, after rapid industrialization and urbanization, air pollution in China has worsened [21]. Air pollution has significantly affected the health of Chinese people as 1 of the top 10 risk factors for death [18]. Several large cohorts confirmed that the particulate matter (PM_2.5_) concentration in the environment is associated with the risk of lung adenocarcinoma in nonsmokers and lung cancer mortality in lifelong nonsmokers [20,22]. When the development of PNA started in 1990, the secondary industry accounted for more than 75% and the tertiary industry accounted for only 20% of the GDP of PNA. In 2018, the status of the secondary and tertiary industries was reversed, and the proportion of the tertiary industry exceeded 75% of the PNA GDP. However, the development of secondary industries cannot prevent air and environmental pollution. A long-period comparative analysis of air pollution in Shanghai analyzed the continuous Morlet wavelet transform on the time series of a 5274-day air pollution index from 2000. The monthly variation in air pollution in Shanghai was not significant, and air pollution in Shanghai showed an increasing trend, but the situation has reversed since 2015 [23]. It is evident that in the past 50 years, especially before 2015, air pollution in PNA has led to an increasing trend in the YLL due to premature death in people with lung cancer in Shanghai.

The initial treatment of lung cancer is relatively simple, including surgery, chemotherapy, and radiotherapy. In the early 2000s, the key genes for lung cancer were identified. In this field, molecular detection is the basic method for guiding and individualizing treatment selection tools. Epidermal growth factor receptor (EGFR) tyrosine kinase inhibitors (TKIs) are small molecule–targeted drugs widely used in advanced non-small-cell lung cancer (NSCLC). Its effectiveness can be shown in symptom improvement, lesion control, and prolongation of progression-free survival (PFS). Women and nonsmoking patients were the dominant groups in TKI treatment [24]. The introduction of immune checkpoint inhibitors in 2015 was an important milestone in the treatment of lung cancer. Immunotherapy has been proven to have a long-lasting positive effect on patients with NSCLC and has been rapidly upgraded to first-line treatment after the success of second-line and backline treatments [25,26]. Since 2003, lung cancer–targeting drugs have entered the scope of Shanghai medical insurance and are widely used in lung cancer treatment. Furthermore, immunotherapy and antiangiogenic drugs have also been gradually introduced in Shanghai. The diversity of drug selection and the individualization and accuracy of treatment schemes directly affect the survival of patients with lung cancer. This may explain why the YLL rate showed a downward trend with an AAPC of the total population after 2005 compared with that before 2005. We found that, except for patients over 80 years old, the CMRs and YLL rate decreased after 2000 in almost all age groups. The changes in these data are related to the rapid development of tumor-targeted therapy and immunotherapy over the past 20 years.

Lung cancer is the leading cause of cancer-related deaths worldwide, with 2,206,771 new lung cancer cases and 1,796,144 deaths in 2020. Lung cancer has a high incidence rate and contributes to 30% of all cancer-related deaths in China. The mortality trends in lung cancer in the United States have gradually decreased [27]. Mortality rates increased from 1990 by 3.91% per year and decreased from 2004 by 1.95% in Montenegro [28]. Some studies have observed a sharp increase in the lung cancer mortality rate since 2000 in China [27]. The lung cancer mortality rate increased from 30.18% in 2004 to 36.10% in 2010 [29]. In 2018, the mortality rate of Chinese men was 68% higher than their US counterparts, while that of Chinese women was similar to that of US women. The difference in men may be related to smoking [27]. Lung cancer mortality in China may increase by 40% between 2015 and 2030 [30]. Our study also found that mortality increased significantly in both genders in PNA. In addition to reducing the proportion of smokers, the widespread application of chest computerized tomography (CT) screening will impact lung cancer mortality in China [27].

### Strengths and Limitations

This study has major strengths, including a large population size (over 8 million) and a relatively long time span (47 years). However, there are several limitations to the study. First, all our data were from a single district, although this district is the largest in Shanghai. Second, there were no data on lifestyle, histological typing, and disease history in our study, so it was impossible to determine the role of each risk factor that may lead to changes in lung cancer mortality [31]. Nonetheless, our study was based on complete and accurate public data over 4 decades from the government surveillance system, and high data quality was ensured.

### Conclusion

This population-based study found increasing trends in the mortality and burden of lung cancer in men and women, and the total population in a developed region of China from 1973 to 2019. Demographic factors, particularly aging, contributed to an increase in mortality. Our study can contribute to a better understanding of lung cancer and can be used in similar cities to design future prevention plans.

## Appendix

Multimedia Appendix 1 Trends in the proportion of the ≥65-year age group in the total and female population in Shanghai PNA and the capital per GDP in Shanghai and Shanghai PNA from 1995 to 2018. GDP: gross domestic product; PNA: Pudong New Area.

Multimedia Appendix 2 Age composition of the population in Shanghai PNA from 1973 to 2019. PNA: Pudong New Area.

## Abbreviations

AAPC: average annual percentage change
ASMRW: age-standardized mortality rate by Segi’s world standard population
CMR: crude mortality rate
GDP: gross domestic product
ICD-10: International Classification of Diseases 10th Revision
NSCLC: non-small-cell lung cancer
PNA: Pudong New Area
TKI: tyrosine kinase inhibitor
YLL: years of life lost

## Data Availability

The data that support the findings of this study are available from the Center for Disease Control and Prevention (CDC) of Pudong New Area (PNA), Shanghai, but restrictions apply to the availability of these data, which were used under license for this study; therefore, they are not publicly available. However, data are available from the authors upon reasonable request and with permission from the CDC of PNA.
