# Supplementary figures and images for: Long-term Changes in the Premature Death Rate in Lung Cancer in a Developed Region of China: Population-based Study
Source: JMIR Public Health Surveill. 2022 Apr 20;8(4):e33633. doi: 10.2196/33633 (PMC9069300; doi:10.2196/33633)

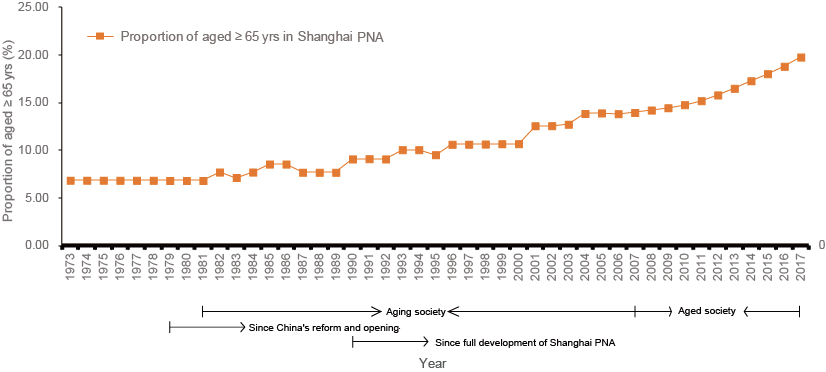

Supplement: Multimedia Appendix 1 [file publichealth_v8i4e33633_app1.png]

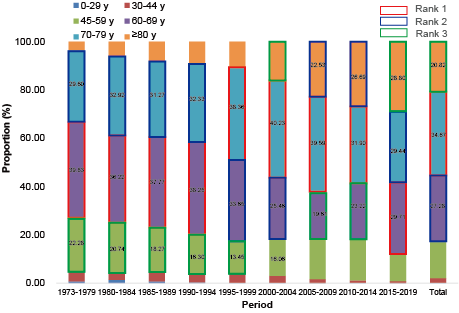

Supplement: Multimedia Appendix 2 [file publichealth_v8i4e33633_app2.png]
